# A QTL on chromosome IV explains a natural variation of QR.pap final position in *Caenorhabditis elegans*

**DOI:** 10.17912/micropub.biology.000836

**Published:** 2023-05-19

**Authors:** Clément Dubois, Marie-Anne Félix

**Affiliations:** 1 Institut de Biologie de l'École Normale Supérieure, Paris, Île-de-France, France

## Abstract

In *Caenorhabditis elegans* , the QR neuroblast and its progeny migrate from the posterior to the anterior part of the animal during the L1 stage. We previously showed that the final position of QR.pa daughters varies among *C. elegans* wild isolates, with CB4932 displaying a particularly anterior QR.pap position (Dubois et al., 2021). Here, we study the genetic basis of the variation between isolates CB4932 and JU1242. We show that JU1242 alleles behave in a mostly dominant fashion. Using a Bulk Segregant Analysis, we detect a quantitative trait locus (QTL) region on chromosome IV. This QTL was confirmed using reciprocal chromosome IV introgressions.

**Figure 1. A main QTL on chromosome IV underlies the difference in QR.pap final position between CB4932 and JU1242:**
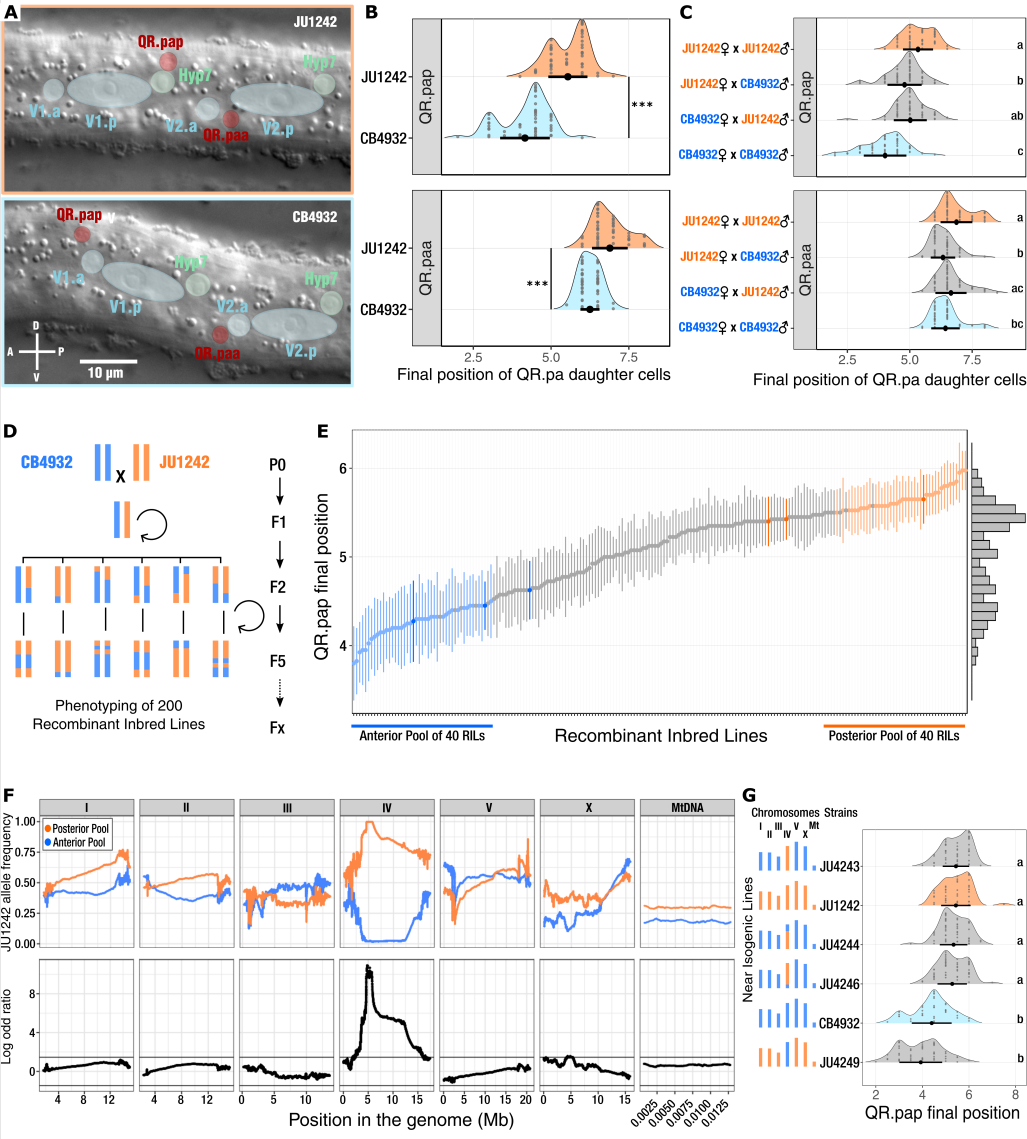
**(A)** Nomarski micrographs of late L1 larvae showing examples of QR.paa and QR.pap positions in JU1242 (top) and CB4932 (bottom). **(B)** Distribution of QR.paa and QR.pap positions in JU1242 and CB4932, replotted from data in Dubois et al., 2021. n=50 per line. Large dots and error bars represent the mean ± s.d. Statistical differences were assessed using a Wilcoxon’s rank sum test (***p-value<0.001). **(C)** Distribution of QR.paa and QR.pap positions in the F1 progeny of JU1242 and CB4932. n=50 per condition. Letters (a-d) represent groups with similar mean (Dunn’s test with Bonferroni adjustment post-hoc comparison). For instance, "ab" means that this condition is not significatively different from either "a" or "b", which are different from each other. **(D)** Generation of Recombinant Inbred Lines (RILs). A single pair of chromosomes is represented for clarity. **(E)** Distribution of QR.pap final position in the 200 RILs. The parental genotypes CB4932 (dark blue) and JU1242 (dark orange) were phenotyped independently three times. The 40 RILs with the most anterior QR.pap position (light blue) were pooled for sequencing, as well as the 40 with the most posterior position (light orange). Dots and error bars represent the mean ± c.i. (95%); n=20 per line. A histogram of the phenotypic distribution is shown on the right. **(F)** Bulk Segregant Analysis: JU1242 allele frequency in the Posterior pool (orange) and the Anterior Pool (Blue). Thin horizontal black lines represent the threshold for a significant difference between log-odd ratios at p = 0.001. The JU1242 allele frequency is fixed in the Posterior Pool between 4.5 Mb to 6 Mb, revealing a QTL associated to the variation in QR.pap final position. **(G)** Validation of the chromosome IV effect on QR.pap final position. Near isogenic Lines were generated using successive backcrosses to exchange chromosome IV between CB4932 and JU1242. The chromosomes from CB4932 are represented in Blue and those from JU1242 in Orange. n>40 scored animals per line. Dots and error bars represent the mean ± s.d. Letters (a-c) represent groups of genotypes with a similar QR.pap mean (Dunn’s test with Bonferroni adjustment post-hoc comparison).

## Description

**CB4932 and JU1242 exhibit a strong difference in QR.pap final position** . During the L1 stage, the QR neuroblast migrates at a long range while undergoing three rounds of division (Sulston and Horvitz, 1977) . Its granddaughter QR.pa stops migration upon expressing *mig-1* , encoding a Wnt receptor, and divides (Mentink et al., 2014) . Thereafter, its QR.paa daughter migrates a short distance posteriorly and ventrally, whereas QR.pap migrates anteriorly and dorsally. The mean position of QR.paa and QR.pap, collectively called QR.pax, is used to quantify the end position of QR.pa migration (Ch’ng et al., 2003; Harris et al., 1996; Mentink et al., 2014; Whangbo and Kenyon, 1999).

Using a set of 40 isolates, we previously showed that *C. elegans* wild isolates present variation in QR.pax final position (Dubois et al., 2021) . We focused on two genetically close (Cook et al., 2017) isolates, JU1242 and CB4932 , which present a strong difference in QR.paa and QR.pap final position ( **Figure 1A,B** ). This difference is mainly due to QR.pap position being far more anterior in CB4932 than in JU1242 (mean comparison: 4.16 vs 5.54, W=207.5 , p-value < 10 ^-12^ ). QR.paa is also significantly more anterior in CB4932 , albeit with a smaller difference (6.25 vs 6.89, W=432, p-value < 10 ^-8^ ) ( **Figure 1B, Ext . Data Table 1** ).

**F1 hybrids indicate that JU1242 alleles act in a mostly dominant fashion for QR.pap position.** In order to investigate the genetic basis of the variation in QR.pap final position between JU1242 and CB4932 , we performed laboratory crosses. We first tested the phenotypes of F1 heterozygous animals. To do so, old, sperm-depleted, hermaphrodites of JU1242 and CB4932 were crossed to males of the opposite genotype. We phenotyped F1 hermaphrodites, as determined by the presence of the hermaphrodite-specific neurons (HSN). The mean final position of QR.pap in heterozygotes is closer to that of JU1242 homozygotes ( **Figure 1C, Ext . Data Table 2** ), and is significantly different from both parents in the case of the cross with CB4932 males but not in the reverse cross (5.02 vs 5.33, Z=1.97, p-value = 0.15) **.** Overall, this suggests that the CB4932 allele at the main QTL on chromosome IV is recessive or weakly semi-dominant for the QR.pap phenotype.

**One main QTL on chromosome IV underlies the variation in QR.pap final position** . We then used a Bulk Segregant Analysis (BSA) approach combined with whole genome sequencing. The main purpose of this method is to find genomic regions associated with a difference of phenotype between two parental genotypes. This method was first developed in plants (Michelmore et al., 1991) and is now commonly used in association with whole-genome sequencing in a broad range of organisms including arthropods (Kurlovs et al., 2019) , yeasts (Birkeland et al., 2010; Parts et al., 2011) and nematodes (Doitsidou et al., 2010; Frézal et al., 2018).

To this end, we generated 200 Recombinant Inbred Lines (RILs) by crossing CB4932 and JU1242 . We singled the progeny at each generation until the fifth generation ( **Figure 1D** ). We then measured the final position of QR.paa and QR.pap in the RILs from the 6 ^th^ generation. The two parental genotypes were phenotyped three times during the scoring of the RILs ( **Figure 1E, Ext . Data Table 3** ).

The two parental lines and two pools of 40 RILs with the most contrasted phenotypes ('Anterior Pool' and 'Posterior Pool') ( **Figure 1E** ) were whole-genome sequenced and the JU1242 allele frequency plotted for each pool ( **Figure 1F** ). A highly significant QTL peak was found on chromosome IV. Indeed, the allele frequency of JU1242 was found to be fixed between 4.5 Mb and 6 Mb in the Posterior pool, and the CB4932 allele frequency in the Anterior pool ( **Figure 1F** ).

**Chromosome IV introgressions confirm the QTL** . To validate the QTL on chromosome IV, we introgressed this chromosome from JU1242 into the CB4932 background and reciprocally. The introgression of the full or part (at least from 2.03 Mb to 14.6 MB) of JU1242 chromosome IV in the CB4932 background leads to a posterior position of QR.pap ( **Figure 1G, Ext . Data Table 4** ). Indeed, the final positions of QR.pap in strains JU4243, JU4244 and JU4246 are not significantly different from that of JU1242 (p-value = 1). Reciprocally, the chromosome IV of CB4932 into the JU1242 background leads to an anterior position of QR.pap, recapitulating the phenotype in CB4932 ( **Figure 1G, Ext . Data Table 4** ).

**Conclusions.** Using a Bulk Segregant Analysis technique, we demonstrated that we were able to detect the genetic basis of the final positioning of a migrating cell. We found a QTL explaining the difference of QR.pap position between two wild isolates. This QTL was confirmed by the exchange of the chromosome IV of the two strains by introgression and appears to account for most of the difference in QR.pax position between CB4932 and JU1242 . The next step would be to find the candidate allele associated with this difference and understand more closely the mechanism and evolution of final positioning of this migrating cell. Overall, this study establishes the quantitative genetic basis of a substantial QR.pap displacement in a wild isolate.

## Methods

***Caenorhabditis elegans* strains and culture.** *Caenorhabditis elegans* strains were cultured at 20°C on 55 mm diameter Petri dishes filled with NGM, and fed on *Escherichia coli* OP50 according to the standard procedures (Brenner, 1974) . We used in this study two wild isolates: JU1242 isolated in Santeuil in October 2007 (Andersen et al., 2012) and CB4932 isolated in Taunton, Great Britain, in January 1991 (Grewal and Richardson, 1991) . The Near Isogenic lines JU4243 *(mfIR126)* , JU4244 *(mfIR127)* , JU4246 *(mfIR129)* and JU4249 *(mfIR132)* were generated during this study ( **Table 1** ).

**QR.paa and QR.pap final position measurements.** The quasi-invariance of *C. elegans* cell lineage and development allows for identification of cells, including the QR.paa and QR.pap neurons (Sulston and Horvitz, 1977; Figure 1A ). To score their position, cultures were roughly synchronized by washing away larvae and adults on plates containing unhatched embryos the day before scoring. The embryos stick to the plate and this procedure allows to obtain late L1 larvae the following morning. For scoring, the larvae were mounted on 3% agar pads with 1 mM sodium azide and observed with a 100x objective using Nomarski optics, as described (Harris et al. 1996, Dubois et al., 2021) . We measured the final position of QR.paa and QR.pap relative to the V lateral epidermal seam cells ( Figure 1A ). To this end, we scored on each mounted slide those late L1 larvae that were at a stage after the first division and before the second division of the V seam cells. From the anterior to the posterior, the V seam cell nuclei form at this stage a constant pattern that we used to generate a relative scale from 0 to 27 (Dubois et al., 2021) . QR.paa and QR.pap have reached their final position before this stage (Sulston and Horvitz, 1977; Harris et al. 1996) .

**Test of dominance.** CB4932 and JU1242 hermaphrodites were aged until 4 days after the L4/adult transition. At this time, the sperm stock of hermaphrodites was depleted and animals laid unfertilized oocytes. Males from the same or the different genotype were crossed with these old hermaphrodites, ensuring heterozygote progenies. Only hermaphrodite L1 larvae were scored, as assessed by the presence of the HSN neuron, specific to hermaphrodites. The four crosses were performed and phenotyped at the same time. The pairwise comparisons between crosses were performed with the Dunn’s test and Bonferroni adjustment to account for multiple testing.

**Construction of Recombinant Inbred Lines.** The parental lines CB4932 and JU1242 were crossed using four males and three hermaphrodites per plate in each direction of the cross (eight plates per direction). 64 F1 individuals were singled and genotyped after laying with the primer pair *par1delF-R* (a 139bp deletion in *par-1* of JU1242 , **Ext. Data Table 5** ). From 19 heterozygous F1 animals, 308 F2 animals were singled. For each line, a single worm was transferred at each generation until the F5 generation, in which most of the genome is assumed to be homozygous. We phenotyped 200 Recombinant Inbred Lines from the 6 ^th^ generation by measuring QR.paa and QR.pap final position in 20 animals per line. The parental phenotypes were scored three times independently during this step.

**DNA extraction and sequencing.** After phenotyping ( **Ext. Data Table 3** ), we collected animals from 40 RILs with anterior QR.pap position (Anterior Pool), and animals from 40 RILs with posterior position (Posterior Pool). RILs with animals showing "abnormal" migration patterns (especially in the dorsoventral position of QR.pax or anteroposterior position of other neurons such as BDU and ALM; Dubois et al. 2021) were not included in these pools. The RILs were then discarded. Genomic DNA of the two parents and the two pools were extracted (Qiagen Puregene Core® kit A) and sequenced by Illumina with 2x150bp paired-end reads, and 20 million reads in total, representing a mean coverage of 20x. Library preparation and whole genome sequencing were performed by the Eurofins Genomics company. The reads are available at NCBI SRA under BioProject PRJNA956481.

**Genomic analysis of the parental strains.** The genomic analysis was performed using the first seven steps of the mapping-by-sequencing pipeline from Besnard et al., 2017. Briefly, reads were mapped to the *C. elegans* reference N2 genome (WS274 genome version ftp://ftp.wormbase.org/pub/wormbase/releases/WS274/species/c_elegans/PRJNA13758/c_elegans.PRJNA13758.WS274.genomic.fa.gz) with bowtie2 (version 2.3.5.1, Langmead and Salzberg, 2012) and the --sensitive preset. The read-group information was added and the duplicated reads were marked and removed by Picard (Version 2.21.3, Broad Institute). The file was then indexed with Samtools (Version 1.9, Li et al., 2009) and filtered with BQSR by bootstrapping a first call made with HaplotypeCaller function from GATK tool suite (Version 4.1.4.0, Auwera et al., 2013). Then, a single .vcf file containing the different SNPs of the two parental genotypes was created. Bad quality (QUAL<20) and heterozygous SNPs were filtered out with VariantFiltration and SelectVariant functions from the GATK tool suite. Large insertions, inversions and deletions were detected with Pindel (version 0.2.5b9, Ye et al., 2009). We annotated SNPs with the variant predictor software SNPeff (Version 4.3t, Cingolani et al., 2012).

**Bulk Segregant Analysis.** The genomes of the Anterior pool and the Posterior pool were mapped to the reference genome as previously described, and a variant calling was performed using GATK tool suite giving the list of variants of JU1242 and CB4932 as predefined positions. We selected only SNPs with a coverage higher than ten in each pool. At each position, we calculated the JU1242 allele frequency by dividing the number of reads of the alternative allele by the total number of reads. If variants were from CB4932 , we calculated the JU1242 allele frequency by 1-(readVar/readTot). We then used a sliding window approach in order to smooth the allele frequencies, with a window of 200 bp and a step of 1 bp. To test whether the allele frequencies of the two pools differed, we calculated the log-odds ratio at each position as previously described in Frézal et al., 2018. Briefly, we calculated the log-odds ratio as:

$$log\left(\frac{\left(\frac{m1+0.000001}{40-m1}\right)+\mathrm{\&nbsp;}0.000001}{\left(\frac{m2+0.000001}{40-m2}\right)+\mathrm{\&nbsp;}0.000001}\right)$$

where m1 and m2 are JU1242 allele frequency in the Posterior pool and the Anterior pool, respectively, multiplied by the number of RILs in each pool. As the number of RILs in each pool is the same (40), we added 0.000001 to avoid infinite values. To define the threshold of significance (p=0.001), we simulated log-odds ratio for one million draws on a binomial distribution of the two pools.

**Generation and Phenotyping of Near Isogenic Lines.** The chromosome IV of JU1242 was introgressed in the CB4932 background and reciprocally. To do so, we first crossed CB4932 hermaphrodites with JU1242 males. Then, we backcrossed males from the F1 progeny with CB4832 hermaphrodites. We singled the F2 progenies and let them self for one generation. If the chromosome IV was from JU1242 , we then backcrossed again F3 progenies two times with CB4932 males and let the F5 self. If the chromosome IV at the F2 was still CB4932 , we backcrossed it with JU1242 males. The male progenies were then crossed with JU1242 hermaphrodites to introgress the Chromosome IV of CB4932 in the JU1242 background. During the process of selfing, the six chromosomes were followed ( **Ext. Data Table 6** ) with the primers described in **Ext. Data Table 5** . The pairwise comparisons of the phenotypes between lines were performed with the Dunn’s test and Bonferroni adjustment to account for multiple testing.

**Statistical analyses, plots and raw data.** Statistical analyses and plots were performed using R version 3.5.2 (R core Team, 2018) , RStudio version 1.1.463 (RStudio Team, 2015) and the following packages: Rmisc (Hope, 2013) , ggplot2 (Wickham, 2016) , ggstance (Henry et al., 2019) , dplyr (Wickham et al., 2020) and dunn.test (Dinno 2017) . The distribution of QR.paa, QR.pap and QR.pax final positions in Figures 1 B, C and G were represented with ggridges (Wilke, 2018) using a bandwidth of 0.25 for smoothing.

## Reagents

***C. elegans* strains used in this study**

**Table:** 

**Strain**	**Genotype**	**Description**	**Source**
CB4932	wild isolate	Taunton, Great Britain,in January 1991 (Grewal and Richardson, 1991)	CGC
JU1242	wild isolate	Santeuil, France, in October 2007 (Andersen et al., 2012)	Our lab
JU4243	*mfIR126*	Introgression of JU1242 chrIV into CB4932 background (see **Ext. Data Table 6** for genotyping data)	This study
JU4244	*mfIR127*	Introgression of JU1242 chrIV into CB4932 background (see **Ext. Data Table 6** for genotyping data)	This study
JU4246	*mfIR129*	Introgression of JU1242 chrIV into CB4932 background (see **Ext. Data Table 6** for genotyping data)	This study
JU4249	*mfIR132*	Introgression of CB4932 chrIV into JU1242 background (see **Ext. Data Table 6** for genotyping data)	This study

## Extended Data

Description: Supplementary Tables (6 sheets) including the data for QR.pax cell positions used to construct the main figure (Tables S1-4), the primer sequences (Table S5) and genotypes of introgressed lines (Table S6). . Resource Type: Dataset. DOI: 10.22002/vhsr7-sa891
